# Microbial Community of Wilted *Fritillaria ussuriensis* and Biocontrol Effects of *Bacillus tequilensis* and *Trichoderma koningiopsis*

**DOI:** 10.3390/biology13110940

**Published:** 2024-11-17

**Authors:** Hao Wu, Jingjing Lu, Simeng Zhao, Jingyi Fei, Zhimiao Qu, Min Zhao, Hongyan Yang

**Affiliations:** College of Life Sciences, Northeast Forestry University, Harbin 150040, China

**Keywords:** *Fritillaria*, wilt, microbial community, *Bacillus*, *Trichoderma*

## Abstract

The cultivation of *Fritillaria ussuriensis* faces challenges due to the prevalent *Fritillaria* wilt disease, hindering large-scale production. To address this, we aimed to understand the disease’s characteristics and develop effective prevention measures. Microbial communities of diseased *F. ussuriensis* plants were analyzed, pathogenic and antagonistic strains were screened, and biocontrol feasibility was tested. We identified *Botryotinia* predominance in aboveground parts and variations in *Mrakia*, *Humicola*, *llyonectria*, and *Fusarium* in underground parts. The pathogens *Fusarium oxysporum* IFM-1 and *Fusarium solani* IFM-52 isolated from diseased *F. ussuriensis* not only caused severe *Fritillaria* wilt but were also pathogenic to *Lilium lancifolium* and *Allium cepa* var. *aggregatum* in Liliaceae. The antagonistic *Bacillus tequilensis* LFM-30 and *Trichoderma koningiopsis* IFM-47 isolated from diseased plants significantly alleviated plant wilt and showed promise in preventing wilt disease caused by *Fusarium* in Liliaceae plants. Our study highlights distinct microbial differences between healthy and diseased *F. ussuriensis* and underscores the pathogenicity of *Fusarium*. Using *T. koningiopsis* and *B. tequilensis* either singly or in combination could offer effective biocontrol against *F. solani* and *F. oxysporum*, benefiting *F. ussuriensis* and related Liliaceae plants.

## Simple Summary

*Fritillaria ussuriensis* holds significance as one of the most crucial traditional Chinese medicinal plants utilized in the prevention and treatment of various human diseases. Currently, its availability depends on large-scale cultivation, with wilt disease posing a significant challenge during the planting process. Endophytic microorganisms within the plants play a vital role in maintaining plant health; however, disparities in microbial communities between diseased and healthy plants remain unclear. This study aimed to compare these variances, elucidate the distribution patterns of pathogens, and identify antagonistic microorganisms with biocontrol properties. The research revealed that these microorganisms were effective not only in combatting wilt in *F. ussuriensis* but also in controlling rot in other plants within the Liliaceae family. This discovery offers a promising avenue for the sustainable management and prevention of soil-borne diseases in plants.

## 1. Introduction

*Fritillaria* spp. include approximately 140 species of perennial plants [1], which are widely distributed worldwide [2]. Some *Fritillaria* plants are ornamental, whereas others can be used as medicinal raw materials. The medical history of *Fritillaria* can be traced back more than 2000 years, and today, it is one of the most commonly used traditional Chinese medicines [3]. *Fritillaria ussuriensis* (*F. ussuriensis*) is a representative *Fritillaria* plant. Its main effects include dissipating heat, moistening the lungs, dissolving mucus, and relieving cough [4]. *Fritillaria* bulbs have thousands of years of medical history in China and herbs of *Fritillaria* have a high market value and demand [5]. As its medical value has gradually increased, the market demand for *F. ussuriensis* has concurrently increased [3]. However, wild populations of *F. ussuriensis* have declined sharply due to excessive foraging.

Cultivation of *F. ussuriensis* has been achieved in black soil [6]. Disturbances from soil-borne diseases have become a major threat to the cultivation of *F. ussuriensis* [7]. One of these diseases is *Fritillaria* wilt. When the disease occurs, plant growth essentially stops, and there are obvious signs of dwarfism and yellowing of the stems and leaves. In severe cases, gradual plant withering and dying occurs, and bulbs rot. *F. ussuriensis* often experiences high mortality in large areas, which results in severe reductions in production. However, the mechanisms related to outbreaks of *Fritillaria* wilt are poorly understood.

Recently, an analysis of the microbial communities in diseased and healthy *F. ussuriensis* rhizosphere soils was undertaken via high-throughput sequencing and revealed that the abundance of the fungal taxa *Fusarium* and *Humicola* in diseased soil influences disease severity [6]. However, the microbial distributions of different parts of diseased plants were not explored. According to previous studies, growth-related microbiomes present significant differences in microbial communities between rhizosphere soil and plant [8,9], and the plant endophytic microbiome is a good source for screening biocontrol microorganisms for sustainable *F. ussuriensis* production [10]. Therefore, understanding the *F. ussuriensis* microbiome is essential for sustainable disease control of *Fritillaria* wilt.

Biological control has become a sustainable way to control soil-borne diseases [11]. *Bacillus*, *Pseudomonas*, and *Trichoderma* strains isolated from plants have been widely used for controlling plant wilt disease [12]. However, for soil-borne diseases of *F. ussuriensis*, there are few reports of screening for biological control microorganisms, except *Trichoderma*, which is known to alleviate Sclerotinia disease [13].

In this study, we hypothesized that there are differences in the microbial communities of diseased and healthy plants and investigated microbial community distribution and differences between the two groups of plants. We then isolated biocontrol microorganisms from diseased plants and tested their feasibility for controlling *Fritillaria* wilt to contribute to the sustainable control of *Fritillaria* wilt.

## 2. Results

### 2.1. Analysis of Microbial Diversity and Richness in Diseased and Healthy F. ussuriensis

**Figure 1:**
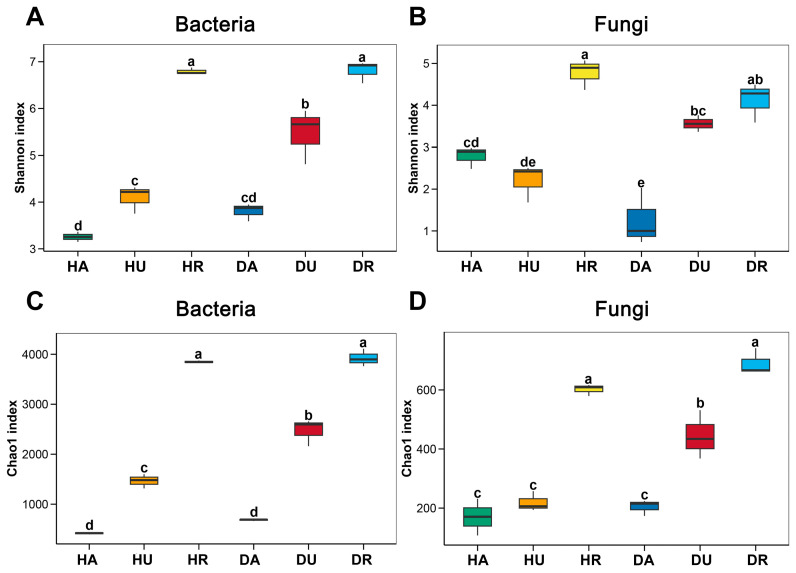
Microbial richness and diversity in healthy and diseased plants and rhizosphere soils. (**A**,**C**) bacteria; (**B**,**D**) fungi. Different colors show differrent groups. HA: healthy aboveground tissue; HU: healthy underground tissue HR: healthy rhizosphere soil; DA: diseased aboveground tissue; DU: diseased underground tissue; DR: diseased rhizosphere soil. Values with different lowercase letters (a–e) above the bars indicate a significant difference (*p* < 0.05).

In this study, the microbiomes of the rhizosphere soil and underground and aboveground parts of *F. ussuriensis* were examined. The results of the Shannon (diversity) and Chao1 (richness) indices are shown in Figure 1. Both bacterial and fungal diversity and richness were greater in the healthy rhizosphere (HR) and diseased rhizosphere (DR) (*p* < 0.05) than in the plants. There were no significant differences between HR and DR. In healthy plants, the bacterial diversity and richness in the plant aboveground parts (HA) were lower than those in the underground parts (HU) (Figure 1A,C), whereas fungal diversity was not significantly different between HA and HU (Figure 1B,D). In diseased plants, bacterial and fungal diversity and richness increased from DA to DU to DR.

### 2.2. Analysis of the Microbial Community Composition

#### 2.2.1. Differences and Similarities in Microbial Community Composition

**Figure 2:**
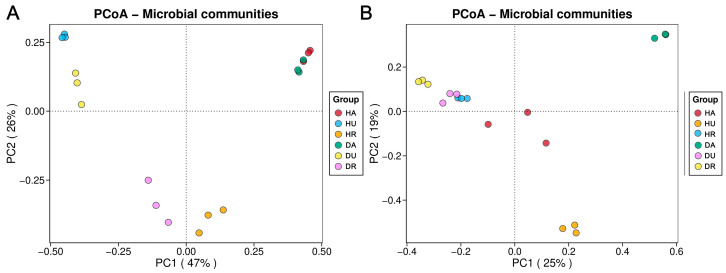
The PCoA of differences in microbial composition between healthy and diseased plants and rhizosphere soil. (**A**) bacteria; (**B**) fungi. HA: healthy aboveground tissue; HU: healthy underground tissue HR: healthy rhizosphere soil; DA: diseased aboveground tissue; DU: diseased underground tissue; DR: diseased rhizosphere soil.

The results of the PCoA based on Bray–Curtis distances (Figure 2) revealed that the first and second principal coordinates explained 47% and 26% of the total variance in the bacterial community composition, respectively. Furthermore, 25% and 19% of the total variance in the fungal community composition was explained by the first and second principal coordinates, respectively. The bacterial communities of the aboveground parts of the plants (HA and DA), underground parts of the plants (HU and DU), and rhizosphere soils (HR and DR) were clearly separated (Figure 2A). HU and DU were separated on the PC1 axis. For the fungal communities, HA and HU were significantly separated from DA and DU (Figure 2B). In the aboveground parts, significant separation mainly occurred. However, HR and DR were not separated. The fungal community in diseased plants (DU) was clustered with those in the DR and HR groups.

#### 2.2.2. Microbial Community Composition in the Diseased Plants and Rhizosphere Soil

**Figure 3:**
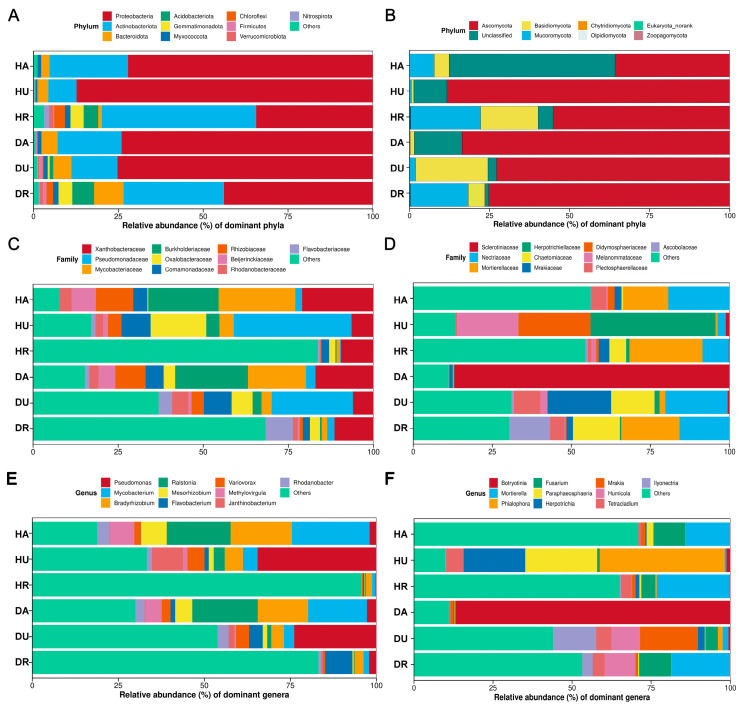
Microbial community composition at phylum, family, and genus level in healthy and diseased groups. (**A**,**C**,**E**) bacteria; (**B**,**D**,**F**) fungi. HA: healthy aboveground tissue; HU: healthy underground tissue HR: healthy rhizosphere soil; DA: diseased aboveground tissue; DU: diseased underground tissue; DR: diseased rhizosphere soil.

For bacteria, at the phylum level, Proteobacteria, Actinobacteria, and Bacteroidota were mainly detected (Figure 3A). Proteobacteria and Actinobacteria were dominant in all the groups. For fungi, Ascomycota, Basidiomycota, and Mucoromycota were mainly detected (Figure 3B). Ascomycota dominated in all the groups. At the family level, the detected bacterial families mainly included *Xanthobacteraceae*, *Pseudomonadaceae*, and *Mycobacteriaceae* (Figure 3C). Fungi mainly included *Sclerotiniaceae*, *Nectriaceae*, and *Mortierellaceae* (Figure 3D).

**Figure 4:**
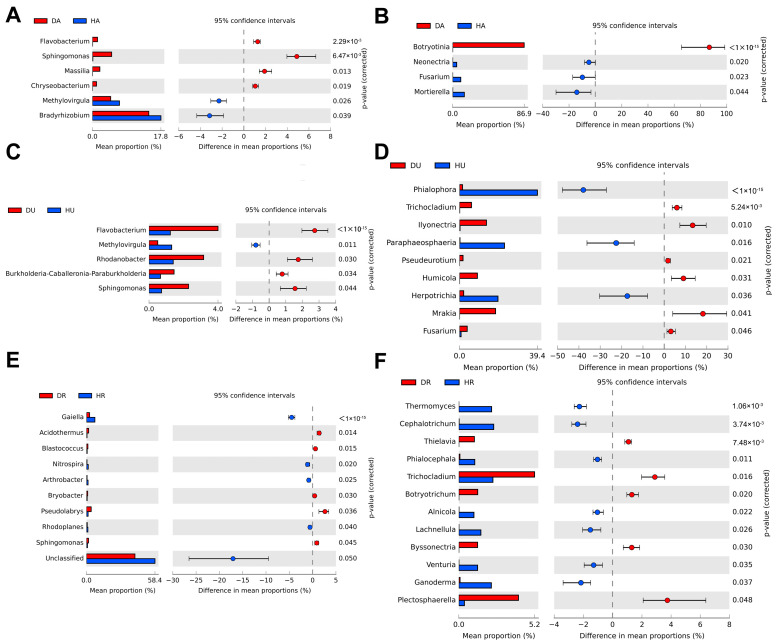
Difference analysis of community composition at genus level in healthy and diseased groups. (**A**,**C**,**E**) bacteria; (**B**,**D**,**F**) fungi. The red or blue colored circle represents the diseased or the healthy group, respectively. HA: healthy aboveground tissue; HU: healthy underground tissue HR: healthy rhizosphere soil; DA: diseased aboveground tissue; DU: diseased underground tissue; DR: diseased rhizosphere soil. The significant level is at *p* < 0.05.

At the genus level, the bacterial genera mainly included *Pseudomonas*, *Mycobacterium*, and *Bradyrhizobium* (Figure 3E), and the fungal genera mainly included *Botryotinia*, *Mortierella*, *Phialophora*, *Fusarium*, and *Paraphaeosphaeria* (Figure 3F). For the bacterial community, in diseased *F. ussuriensis*, the abundances of *Sphingomonas*, *Flavobacterium*, *Massilia*, and *Chryseobacterium* were greater in the aboveground samples than in the healthy control samples (Figure 4A). In *Fritillaria* underground parts, the abundances of *Flavobacterium*, *Rhodanobacter, Sphingomonas*, and *Burkholderia-Caballeronia-Paraburkholderia* were relatively higher (Figure 4C). In the rhizosphere soil, the abundances of *Acidothermus*, *Blastococcus*, *Pseudolabrys*, *Sphingomonas*, and *Bryobacter* were relatively higher (Figure 4E). For the fungal community, in aboveground parts, the abundance of *Botryotinia* was greater in the DA plants (Figure 4B). The underground abundances of *Mrakia*, *Ilyonectria*, *Humicola*, *Trichocladium*, *Fusarium*, and *Pseudeurotium* were relatively higher in DU (Figure 4D). In the rhizosphere soil, the abundances of *Trichocladium*, *Plectosphaerella*, *Botryotrichum*, *Byssonectria*, and *Thielavia* were greater in DR (Figure 4F).

### 2.3. Analysis of Co-Occurrence Networks Based on the Diseased and Healthy Plants and Rhizosphere Soil

**Figure 5:**
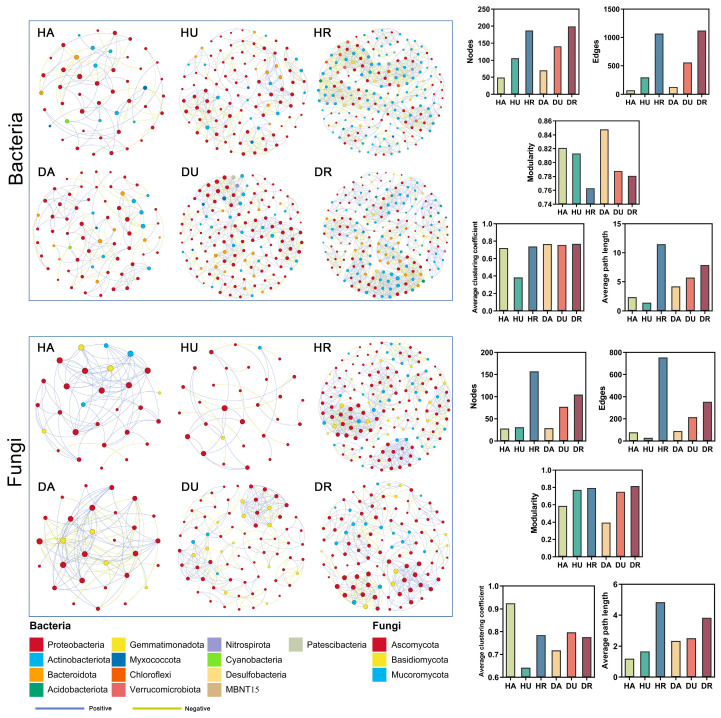
The co-occurrence network analysis of microbial communities in different groups. The colored circles show different fungal or bacterial phyla.

On the basis of the results of the Spearman correlation analysis, the basic topological parameters of the correlation networks were calculated, and co-occurrence networks were constructed on the basis of all the samples (Figure 5). Each node represents an OTU, and each edge represents a strong and significant correlation between the different nodes. For bacteria, the network in the rhizosphere, whether in healthy or in diseased plants, was the most complex (HR and DR), whereas that of the aboveground parts was the simplest (HA). The networks for fungi were simpler than those for bacteria. Similarly, the most complex network occurred in the rhizosphere (HR and DR). The simplest network existed for HU, which was simpler than that for DU.

### 2.4. Isolation and Identification of Pathogenic and Antagonistic Microorganisms

**Figure 6:**
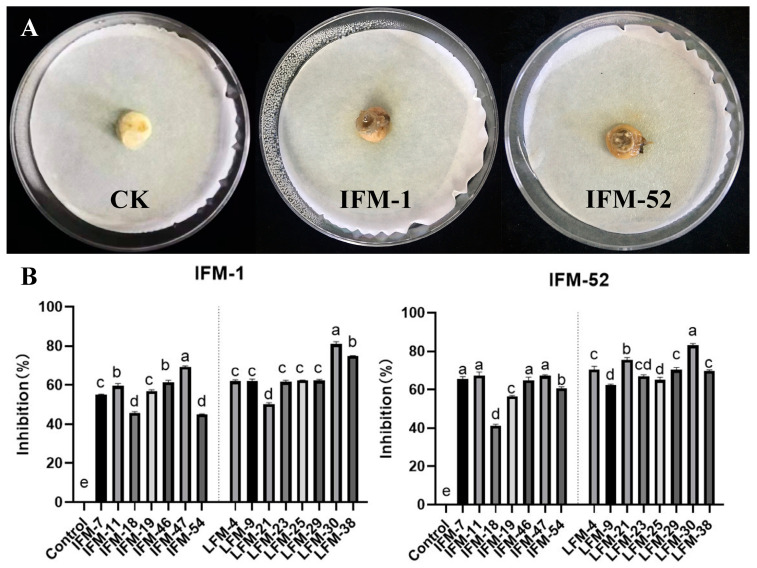
Screening of pathogens and antagonistic microorganisms. (**A**) Pathogens screening; (**B**) the inhibition rate based on plate confrontation. CK: no inoculation; IFM-1: *F. oxysporum*; IFM-52: *F. solani*; IFM-7~IFM-54: antagonistic fungi; LFM-4~LFM-38: antagonistic bacteria. Values with different lowercase letters (a–d) above the bars indicate a significant difference (*p* < 0.05).

To detect potential pathogens on diseased plants, we used diseased plant tissues as materials to isolate 86 fungal isolates (Table S1). Among these 86 isolates, 9 strains were found to be capable of infecting *F. ussuriensis* bulbs. Among them, *Fusarium oxysporum* (*F. oxysporum*) IFM-1 and *Fusarium solani* (*F. solani*) IFM-52 caused the most severe bulk rot (Figure 6A). Fifty-five bacterial and thirty-eight fungal isolates were obtained from healthy *F. ussuriensis* bulbs (Table S1). Using *F. oxysporum* IFM-1 and *F. solani* IFM-52 as target pathogens, plate confrontation experiments were carried out to screen for antagonistic microorganisms. Eight bacterial strains and seven fungal strains with strong antagonistic activity (mycelial inhibition range of 41.26% to 83.00%) against IFM-1 and IFM-52 were obtained (Figure 6B).

**Figure 7:**
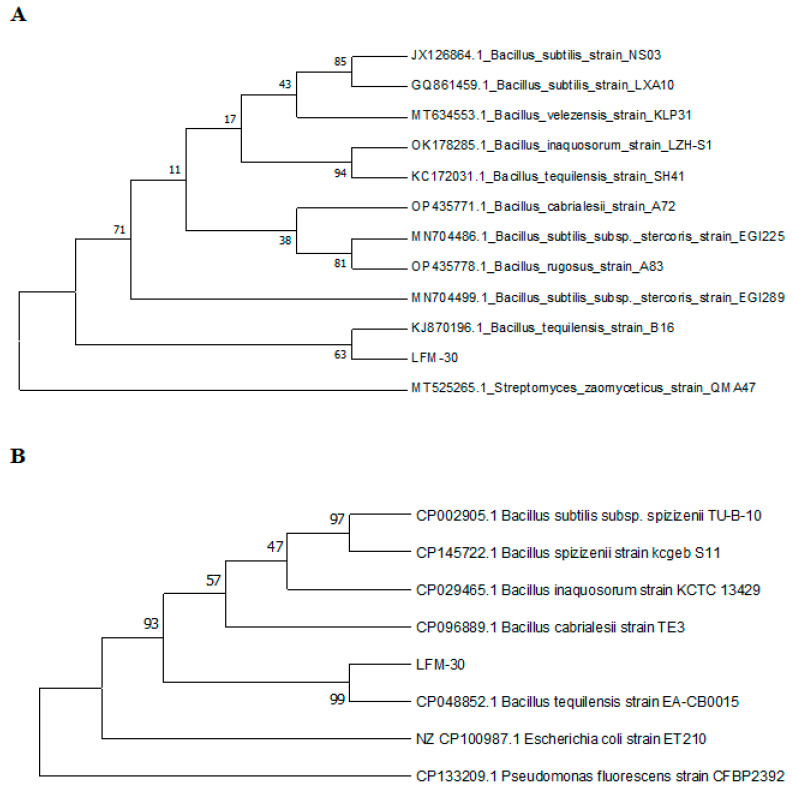
Phylogenetic analysis of LFM-30 16S rRNA (**A**) and gyrB (**B**) gene sequencing using the neighbor-joining method.

**Figure 8:**
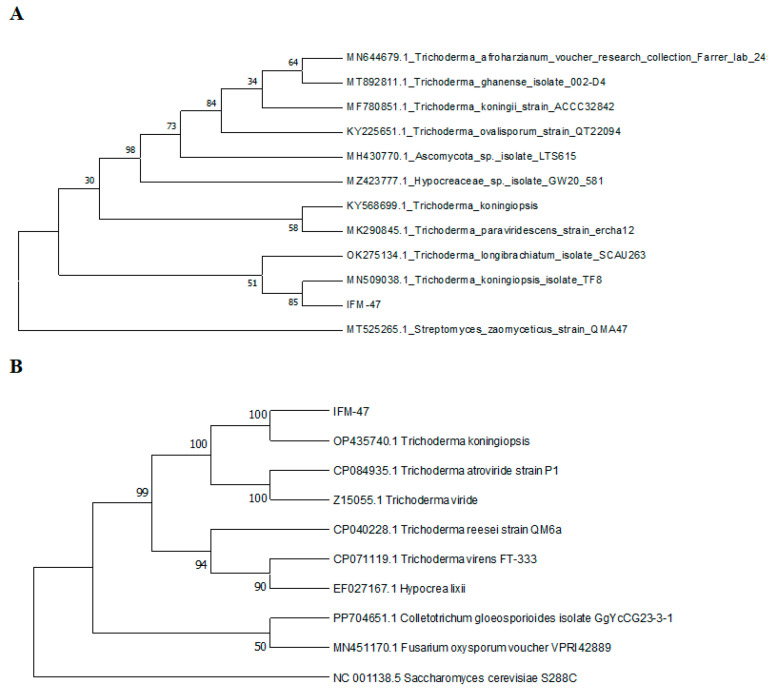
Phylogenetic analysis of IFM-47 ITS (**A**) and β-tubulin (**B**) gene sequencing using the neighbor-joining method.

*Bacillus tequilensis* (*B. tequilensis*) LFM-30 (Figure 7) of the bacteria and *Trichoderma koningiopsis* (*T. koningiopsis*) IFM-47 (Figure 8) of the fungi, both individually and in combination, expressed significant antagonistic effects on *F. oxysporum* IFM-1 and *F. solani* IFM-52. 

**Figure 9:**
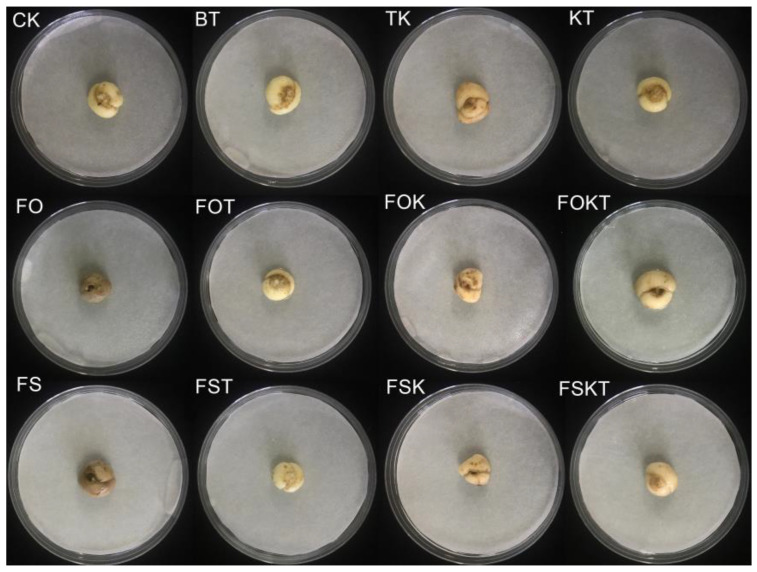
Effects of in vitro *F. ussuriensis* bulb inoculation. CK: no inoculant; BT: *B. tequilensis* LFM-30; TK: *T. koningiopsis* IFM-47; KT: BT + TK; FO: *F. oxysporum* IFM-1; FOT: FO + BT; FOK: FO + TK; FOKT: FO + TK + BT; FS: *F. solani* IFM-52; FST: FS + BT; FSK: FS + TK; FSKT: FS + TK + BT.

Furthermore, the results of in vitro bulb experiments revealed that neither *B. tequilensis* LFM-30 nor *T. koningiopsis* IFM-47 was pathogenic to *F. ussuriensis* bulbs (Figure 9).

### 2.5. Effects of Inoculation Based on Pot Experiments

**Figure 10:**
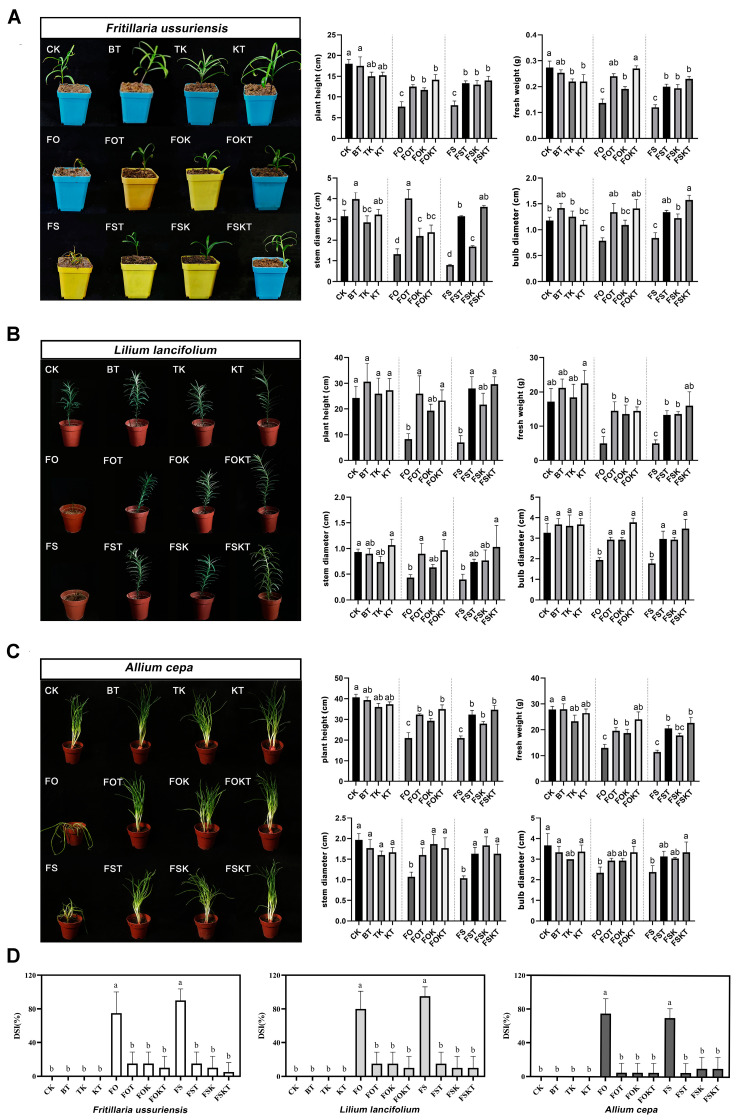
Inoculating effects of antagonistic microorganisms based on pot experiment. (**A**) *F. ussuriensis*; (**B**) *L. lancifolium*; (**C**) *A. cepa*. CK: no inoculant; BT: *B. tequilensis* LFM-30; TK: *T. koningiopsis* IFM-47; KT: BT + TK; FO: *F. oxysporum* IFM-1; FOT: FO + BT; FOK: FO + TK; FOKT: FO + TK + BT; FS: *F. solani* IFM-52; FST: FS + BT; FSK: FS + TK; FSKT: FS + TK + BT. (**D**) The disease severity index (DSI) of the three Liliaceae species. Values with different lowercase letters (a–c) above the bars indicate a significant difference (*p* < 0.05).

To test the control effect and application potential of antagonistic microorganisms, three Liliaceae species, namely, *F. ussuriensis*, *L. lancifolium*, and *A. cepa,* were used in pot experiments. All the plants in the BT, TK, and KT treatments resembled those in the CK treatment and were in a healthy state (Figure 10), which means that inoculation with the antagonistic microbes *B. tequilensis* LFM-30 and *T. koningiopsis* IFM-47 had no negative impact on the growth of the three plants.

The occurrence of plant diseases was more severe in the FO and FS treatments than in the other treatments and manifested as short and yellow plants that withered or died. The incidence of plant disease was significantly lower with single or mixed inoculation of *B. tequilensis* LFM-30 and *T. koningiopsis* IFM-47 than in the FO and FS treatments (Figure 10).

## 3. Discussion

### 3.1. Microbial Diversity and Richness in Diseased F. ussuriensis Plants and Rhizosphere Soil

To reveal the pathogenesis in diseased *F. ussuriensis* plants and rhizosphere soil, the analysis of microbiological characteristics is necessary and important. Regarding the rhizosphere microbiome of *F. ussuriensis*, detailed differences in diseased and healthy fields have been investigated [6]. However, little is known about the characteristics of the endophytic microbiome of *F. ussuriensis* in diseased plants. The root-associated microbiome is influenced mainly by a combination of ecological and environmental factors and by genetic differences among hosts [14]. In this study, we found that in both healthy and diseased fields, the microbial diversity and richness increased from the aboveground parts of the *F. ussuriensis* plants to the underground parts and to the rhizosphere. This result was consistent with those of previous studies and likely occurred due to ecological niche differences [15]. The fungal diversity and richness were greater in the underground parts of diseased plants than in the aboveground parts (Figure 1), indicating that the microbiome in the underground parts was easily affected by the *F. ussuriensis* physiological state.

### 3.2. Microbial Community Compositional Differences and Stability in Diseased and Healthy Groups

PCoA, which is based on Bray–Curtis distances, was used to reveal differences in microbial community composition [16]. In this study, significant differences were detected among the *F. ussuriensis* aboveground parts, underground parts, and rhizosphere soils (Figure 2), which coincided with the microbial diversity and richness results (Figure 1). The bacterial communities aboveground and fungal communities both aboveground and underground showed significant differences (Figure 2), indicating that plant microbial communities are sensitive to variations in biotic and abiotic factors during plant development and that, because of the presence of phytopathogens, significant shifts in plant-assembled microbial communities occur [17]. The analysis of co-occurrence networks also revealed that the microbiome in the rhizosphere was more stable than the plant microbiome (Figure 5). Theoretically, owing to biotic stress, network stability and complexity might decrease in diseased plants and rhizosphere soil [18]. However, in this study, no similar trend appeared, probably due to the plants being in the disease stage at the time of sampling.

### 3.3. Microbial Community Composition in the Diseased Group

The dominant phyla in all the treatments in this study, such as Proteobacteria, Actinobacteria, and Ascomycota, have been widely detected in terrestrial soils and plants [19,20,21]. For bacteria, at the genus level, the abundances of *Sphingomonas*, often a plant probiotic microorganism [22], were greater in the plants and rhizosphere soil of diseased plants (Figure 4). *Flavobacterium*, some species of which are opportunistic plant pathogens [23], was also detected in the aboveground and underground parts. The abundances of *Massilia* [24] and *Chryseobacterium* [25] were greater in the aboveground parts and the abundances of *Rhodanobacter* [26] and *Burkholderia-Caballeronia-Paraburkholderia* [27] were greater in the underground parts, which have often been reported as beneficial microorganisms for plant growth [28]. The abundances of *Acidothermus*, *Blastococcus*, *Pseudolabrys*, and *Bryobacter*, which are related to nutrient cycling [29,30], were relatively high in the rhizosphere soil.

Notably, *Sclerotiniaceae* was extremely abundant in the aboveground parts of the diseased group (DU) (Figure 3D), indicating that the microbial composition was distinct in the aboveground parts of the diseased group when *Fritillaria* wilt occurred. Furthermore, at the genus level, *Botryotinia,* belonging to Sclerotiniaceae, dominated the fungal community (Figure 3F). A previous study revealed that *Botryotinia* can cause diseases such as gray mold [31] or plant spot disease [32]. Here, our results revealed that *Botryotinia* could contribute to *Fritillaria* wilt. This result also revealed that pathogenic *Botryotinia* were more likely to be obtained from the aboveground parts of plants. *Mrakia*, *Ilyonectria*, *Humicola*, *Trichocladium*, *Fusarium*, and *Pseudeurotium* were more abundant in the underground regions of diseased plants than in healthy plants (Figure 3F). Microbes such as *Mrakia*, *Ilyonectria*, *Humicola*, and *Fusarium* have been reported as pathogenic microorganisms, suggesting that disease occurrence might result from the combined effects of two or more pathogenic genera [33]. Jiao reported that *Humicola* and *Fusarium* in the rhizosphere are indicators of *Fritillaria* wilt [6]. In this study, although we did not detect distinct changes in the above two species in the rhizosphere soil, we detected significant changes in the *Fritillaria* underground regions. Meng found that the rhizosphere soil of plants infected with root rot contained more potential bacterial antagonists than the rhizosphere soil of healthy plants [34]. The results of this study revealed that, in *Fritillaria* underground and rhizosphere soils, pathogenic, probiotic, and functionally unknown microorganisms were accumulated. Therefore, diseased plants and rhizosphere soils could also be used for screening probiotic microorganisms [34].

### 3.4. Isolation of Pathogenic and Antagonistic Microorganisms from the Diseased Tissues

According to previous studies [6] and the results of this research, fungi are one of the main causes of *Fritillaria* wilt. Therefore, we focused on fungal isolation. Among the 86 fungal strains isolated from diseased tissues in this study, 38 strains belonged to *Fusarium*, accounting for 44.2% of the total (Table S1). The combined results of plate inoculation and pot experiments revealed that the *Fusarium* strains isolated in this study were strongly pathogenic not only to *F. ussuriensis* but also to *L. lancifolium* and *A. cepa* in Liliaceae (Figure 6, Figure 9 and Figure 10), which was consistent with the results of previous studies in which *Fusarium* was shown to infect lily [35], onion [36], and *F. przewalskii* [37]. In fact, *Fusarium* not only infects Liliaceae plants but also causes wilt rot or bulb rot diseases in more than 150 other plants [38]. The effective prevention of *Fusarium* infections is highly important for the development of sustainable agriculture.

### 3.5. Application Potential of Antagonistic Microorganisms

The use of biocontrol microorganisms to control root or bulb rot caused by *Fusarium* has been reported for many plants. For *Fritillaria*, *Bacillus subtilis* has been reported to resist bulb rot in *F. taipeiensis* [39] and *F. thunbergii* [40]. For *F. ussuriensis*, *Trichoderma* were revealed to be able to inhibit infection from sclerotinic pathogens [13]. In this study, Liliaceae plants were inoculated with *B. tequilensis* LFM-30 and/or *T. koningiopsis* IFM-47. The results showed that either single or mixed inoculation can help plants resist disease (Figure 9 and Figure 10). It has been reported that *T. koningiopsis* YIM ph30002, which was isolated from 2-year-old healthy *Panax notoginseng*, can antagonize plant pathogens through an antimicrobial mechanism which involves covering plant pathogen colonies through rapid growth, destroying the mycelia of the pathogen, producing volatile organic compounds, and significantly inhibiting the growth of *Fusarium* [41]. It has also been reported that *T. koningiopsis* Tri-41 can effectively parasitize and lyse the mycelia of *F. oxysporum*, the pathogen causing cucumber wilt [42]. Therefore, the biocontrol functions of *T. koningiopsis* IFM-47 might involve the synthetical mechanisms to resist *Fusarium*.

*Bacillus* resistance to *Fusarium* has been reported in medicinal plants. Research on *Rehmannia glutinosa* revealed that *Bacillus* DU-1 can inhibit the growth of *F. oxysporum* and promote plant growth, effectively mitigating challenges associated with the continuous cultivation of *R. glutinosa* [43]. *B. velezensis* can help suppress *F. oxysporum* infection in ginseng [9]. *B. tequilensis* has been reported to help plants resist *Fusarium* on tomatoes and *Angelica dahurica* [44,45]. The mixed application of *T. atrovide* and *B. amyloliquefaciens* has been shown to help wheat resist *F. graminearum* and prevent wheat root rot [46]. The results of these studies demonstrated for the first time that *T. koningiopsis* and *B. tequilensis* isolated from *F. ussuriensis* can be used alone or in combination for the biological control of Liliaceae plant wilt caused by *F. solani* and *F. oxysporum.* Therefore, *T. koningiopsis* and *B. tequilensis* have the potential to control *Fusarium* wilt in Liliaceae plants.

## 4. Conclusions

This study aimed to analyze the microbial population characteristics of diseased *Fritillaria ussuriensis*, screen for pathogenic and antagonistic strains, and test the biocontrol feasibility of antagonistic microorganisms. Predominantly, we observed the prevalence of *Botryotinia* in the aboveground parts, while variations in *Mrakia*, *Humicola*, *llyonectria*, and *Fusarium* were found in the underground parts. The identified pathogens, *Fusarium oxysporum* and *Fusarium solani*, not only induced severe *Fritillaria* wilt but were also pathogenic to other plants in the *Liliaceae* family. However, we discovered that the antagonistic microorganisms *Bacillus tequilensis* LFM-30 and *Trichoderma koningiopsis* IFM-47 exhibited promising effects in alleviating plant wilt and preventing wilt disease caused by *Fusarium* in Liliaceae plants. This research provides a sustainable solution for combating wilt disease in plants.

## 5. Materials and Methods

### 5.1. Plant and Soil Sampling in the Diseased Field

**Figure 11:**
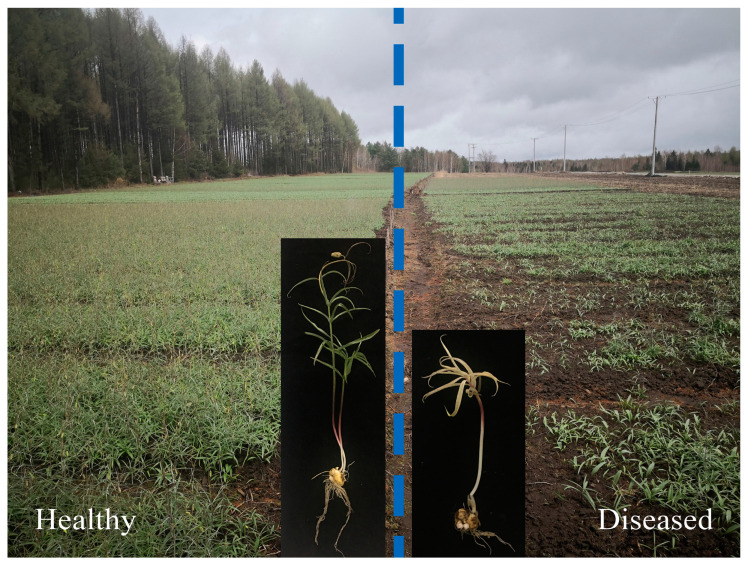
Growth status of *F. ussuriensis* at the planting site while sampling. The left side of the blue dotted line shows the healthy field, and the right side shows the diseased field.

Samples of *F. ussuriensis* and rhizosphere soil were collected from the *F. ussuriensis* planting site of the HongXing Forestry Bureau in Yichun City, Heilongjiang Province (48° 25′ N, 129° 39′ E), on 10 May 2021. Both naturally diseased and healthy fields were located in the same area. Ten *F. ussuriensis* plants with disease symptoms in the diseased field and ten plants without any typical sign of disease in the healthy field were collected (Figure 11). Simultaneously, rhizosphere soil samples were also collected [47]. Upon collection, all samples were promptly stored in ice boxes and transported to the laboratory for further processing, storage, and subsequent analysis.

For *F. ussuriensis*, the plants were carefully washed with running water for 1 h and then separated into underground and aboveground parts. To ensure sterility, plant tissues underwent a sequential sterilization process: immersion in 75% (*v*/*v*) ethanol for 10 s, exposure to a 4% (*v*/*v*) solution of sodium hypochlorite for 30 s, and five rinses with sterile water [48]. Subsequently, the plant tissues were dried on aluminum foil to isolate endophytic microorganisms, or quickly frozen in liquid nitrogen and stored at −80 °C for endophytic microbiome analysis. In this study, rhizosphere soil refers to the soil adhering to the bulb obtained by uprooting the plants, gently shaking off excess soil, and then scraping it with a spoon and storing it at −80 °C for soil microbiome analysis [47]. The samples were labeled as follows: healthy aboveground tissue (HA), healthy underground tissue (HU), healthy rhizosphere soil (HR), diseased aboveground tissue (DA), diseased underground tissue (DU), and diseased rhizosphere soil (DR).

### 5.2. High-Throughput Illumina Sequencing

Microbial DNA was extracted from both rhizosphere soil and plant samples using the E.Z.N.A.^®^ Soil and Tissue DNA Kit (Omega Bio-tek, Norcross, GA, USA) following the manufacturer’s protocols. For bacterial 16S ribosomal RNA gene analysis, the V4-V5 region was amplified by PCR using the primers 515F (5′-barcode-GTGCCAGCMGCCGCGG)-3′ and 907R (5′-CCGTCAATTCMTTTRAGTTT-3′) [49]. A PCR process of 25 cycles involving initial denaturation at 95 °C for 2 min, denaturation at 95 °C for 30 s, annealing at 55 °C for 30 s, extension at 72 °C for 30 s, and a final extension at 72 °C for 5 min was performed. For fungi ribosomal RNA gene analysis, the internally transcribed spacer (ITS) region was amplified using the primers ITS1F (5′-barcode-CTTGGTCATTTAGAGGAAGTAA-3′) and ITS2R (5′-GCTGCGTTCTTCATCGATGC-3′) [47]. PCRs were carried out in triplicate 20 μL mixtures containing 4 μL of 5 × FastPfu Buffer, 2 μL of 2.5 mM dNTPs, 0.8 μL of each primer (5 μM), 0.4 μL of FastPfu Polymerase, and 10 ng of template DNA. Amplicons were extracted from 2% agarose gels and purified using the AxyPrep DNA Gel Extraction Kit (Axygen Biosciences, Union city, CA, USA).

The purified PCR products were quantified with the Life Qubit^®^ 3.0 (Invitrogen, Carlsbad, CA, USA). Amplicons from twenty-four different barcodes were mixed in equal amounts. The pooled DNA product was used for constructing an Illumina Pair-End library following the genomic DNA library preparation procedure of Illumina. Subsequently, the amplicon library was subjected to paired-end sequencing (2 × 250) on an Illumina platform (BIOZERON, Shanghai, China) following standard protocols.

The raw fastq files were initially demultiplexed using in-house perl scripts based on the barcode sequence information for each sample. The following criteria were used: (i) Reads of 250 bp were truncated at any site with an average quality score below 20 over a 10 bp sliding window. Truncated reads shorter than 50 bp were discarded. (ii) Only exact barcode matches were allowed, permitting a maximum of two nucleotide mismatches in primer matches. Reads containing ambiguous characters were removed. (iii) Sequences with an overlap longer than 10 bp were assembled according to their overlap sequence. Reads that could not be assembled were excluded.

Operational taxonomic units (OTUs) were clustered at a 97% similarity cutoff using UPARSE (version 7.1, http://drive5.com/uparse/, accessed on 2 March 2023), and chimeric sequences were identified and removed using UCHIME (http://sourceforge.net/projects/microbiomeutil/files/, accessed on 2 March 2023). The phylogenetic affiliation of each 16S rRNA gene sequence was analyzed using the uclust algorithm (http://www.drive5.com/usearch/manual/uclust_algo.html, accessed on 3 March 2023) against the Silva (SSU138.1) 16S rRNA database with a confidence threshold of 80% [50].

The raw reads from the diseased and healthy groups have been deposited into the NCBI Sequence Read Archive (SRA) database with the accession number PRJNA1055720.

### 5.3. Analysis of Co-Occurrence Network

To investigate the relationship of both bacteria and fungi in the aboveground and underground parts of the plant, as well as in the rhizosphere, a co-occurrence network was constructed based on the Spearman correlation coefficient matrix using NetworkX. Only OTUs with a relative abundance greater than 0.01% in each treatment were selected for network construction. Notably significant interactions were highlighted, with a Spearman correlation threshold set at 0.7, *p* < 0.05. The resulting networks were visually represented using the Gephi v.0.10.1 platform. The topological features, such as average degree and modularity, of the networks were calculated using NetworkX on the Majorbio Cloud Platform (http://www.majorbio.com).

### 5.4. Isolation of Microorganisms and Pathogenicity Test

Diseased and healthy *Fritillaria* bulbs were cut into 0.5 cm × 0.5 cm pieces. The tissue pieces were immersed in 75% ethanol for 10 s, followed by immersion in 4% sodium hypochlorite for disinfection for 30 s, then rinsed five times with sterile water and ground before being spread on LB media for bacterial isolation [51] or PDA medium for fungal isolation [52]. The bacteria were cultured at 37 °C for 2 d, while the fungi were cultured at 28 °C for 5 d. After being streaked twice, the isolates were stored at −80 °C for subsequent experiments.

A pathogenicity test was conducted on *Fritillaria* bulbs. Briefly, bacterial cultures (CFU/mL) or fungal spores (spore/mL) were inoculated with a concentration of 1 × 10^6^ and 20 µL onto the surface-disinfected bulbs [53]. After 2 or 5 d of cultivation, tissues showing obvious disease symptoms (covering > 50% area) were isolated again to observe if the new microorganisms matched the morphology of the initially isolated pathogen. To confirm pathogenicity, the isolated microorganisms were reinoculated into healthy tissue, and those exhibiting consistent symptoms and pathogen morphology were classified as pathogens. The experiment was repeated three times.

The antagonistic performance was assessed using the plate confrontation method [54] with *F. oxysporum* IFM-1 (FO) and *F. solani* IFM-52 (FS), which were screened in this study. After cultivating at 28 °C on agar for 7 d, a pathogen plug was extracted using a 6 mm diameter punch and placed, mycelium side down, onto the center of a fresh PDA plate. A 5 μL overnight culture of bacteria or a plug from 7 d of fungal cultivation was inoculated at the four corners of the confrontation plate, 3 cm from the center. Control plates were prepared with only IFM-1 or IFM-52 placed in the same position. The plates were then incubated at 28 °C for 5 d. The inhibition rate (IE) was calculated using the following formula: IE (%) = (1 − Rc/Rs) × 100%, where Rc represents the radial growth radius of the pathogen in the coculture and Rs represents the radial growth radius of the pathogen in the control.

### 5.5. Identification of Isolates

Genome DNA was extracted from pathogens exhibiting a strong pathogenic phenotype and antagonistic performance. The amplification of DNA was performed using universal primers. For bacterial identification, the primers used were 27F (5′-AGAGTTTGATCCTGGCTCAG-3′) and 1492R(5′-TACCTTGTTACGACTT-3′), targeting the bacterial 16S rDNA gene, as well as the rpoB gene, rpoB-F (5′-AGGTCAACTAGTTCAGTATGGAC-3′), and rpoB-R (5′-AAGAACCGTAACCGGCAACTT-3′) [55]. For fungal identification, the primers ITS1 (5′-TCCTGTAGGTGAACCTGCGG-3′) and ITS4 (5′-TCCTCGCTTATTGATGCC-3′), targeting the fungal rDNA transcription spacer [56], and Bt2a (5′-GGTAACCAAATCGGTGCTGCTTTC-3′) and Bt2b (5′-ACCCTCAGTGTAGTGACCCTTGGC-3′), targeting the *β-tubulin* (*tub2*) gene [57], were employed. PCRs were prepared in 50 μL mixtures containing 15 ng of template DNA, 1 × PCR buffer (Mg^2+^ free), 0.16 mM of each dNTP, 1.5 mM MgCl_2_, 0.45 μM of each primer, and 1 U of Takara rTaq DNA polymerase (TakaRa Ex Taq, Otsu, Japan). The PCR conditions consisted of an initial denaturation at 94 °C for 5 min, followed by 30 cycles of denaturation at 94 °C for 1 min, annealing at 55 °C for 1 min, and elongation at 72 °C for 1 min 30 s, with a final elongation step at 72 °C for 5 min. Subsequently, the amplified product was detected, purified by 1% agarose gel electrophoresis, and subjected to sequencing (Qingke Biotechnology Co., Ltd., Beijing, China). The phylogenetic tree was constructed using MEGA7.0 through the neighbor-joining method [58].

The sequences of the aforementioned isolates were deposited in DDBJ/EMBL/GenBank using the DDBJ Fast Annotation and Submission Tool (DFAST) (https://dfast.ddbj.nig.ac.jp/, accessed on 5 May 2023) and assigned the following accession numbers: IFM-1: PP024275; IFM-47: PP024276; IFM-52: PP024277; LFM-30: PP024283. 

### 5.6. Pot Experiment

#### 5.6.1. Materials

Three species from Liliaceae plant were selected for the pot experiment: *F. ussuriensis* Maxim (5 cm high), *L. lancifolium* (15 cm high), and *A. cepa* var. *aggregatum* (20 cm high). The seedlings of *F. ussuriensis* were transplanted into pots with dimensions of 10 cm (top length), 12 cm (height), and 8 cm (bottom length), with each pot filled with 0.3 kg of soil. *L. lancifolium* and *A. cepa* var. *aggregatum* seedlings were cultivated in pots with a top diameter of 19 cm, a height of 16 cm, and a bottom diameter of 14 cm, each filled with 1.5 kg of soil. After one week of growth, seedlings showing uniform growth were selected for root inoculation experiments. The potting soil used was not autoclaved and had the following characteristics: nitrate nitrogen content of 25.05 mg/kg, ammonium nitrogen content of 0.69 mg/kg, available phosphorus content of 1.18 mg/kg, available potassium content of 292.25 mg/kg, total nitrogen content of 10.2 mg/g, total phosphorus content of 8.59 mg/g, total potassium content of 20 mg/g, organic matter content of 0.35 g/g, and a pH of 6.96.

#### 5.6.2. Preparation of Inoculants for Pot Experiment

The spore suspension of pathogens *F. oxysporum* (FO) and *F. solani* (FS), as well as the antagonistic fungus *T. koningiopsis* IFM-47, was obtained by filtering 7 d liquid cultures at 28 °C and diluting them to 1.0 × 10^8^ spores/mL with sterile water. For the antagonistic bacteria *B. tequilensis* LFM-30, the 2 d liquid cultures at 30 °C were centrifuged at 10,000× *g* for 5 min. The resulting pellet was then diluted to a concentration of 1.0 × 10^8^ CFU/mL using sterile water for inoculation.

#### 5.6.3. Inoculating and Cultivation of *F. ussuriensis*

For each plant, twelve treatments were established, as shown in Table S1. Five replicates were prepared for each treatment. Bacterial or fungal spore suspensions were inoculated through root irrigation. The inoculation rate was calculated as 1 × 10^7^ CFU/g or spore/g of soil. The control (CK) treatment received the same volume of sterile water. After 15 d of inoculation, the growth status of the plants was observed and recorded, and the biomass of the plants was measured through destructive sampling according to the methods described in Section 2.2. The occurrence of bulb rot was evaluated using the bulb rot index. The disease classification criteria were as follows: grade 0 indicated the absence of disease spots on the bulb surface, grade 1 represented a disease area less than 1/10 of the bulb surface area, grade 2 indicated a disease area between 1/10 and 1/3 of the bulb surface area, grade 3 represented a disease area greater than 1/3 of the bulb surface area, and grade 4 indicated a disease area exceeding 2/3 of the bulb surface. The disease index was calculated using the formula described previously [59].

$$DSI(\%)=\frac{\sum \left(Number\;of\;disease\;at\;all\;levels\times Relative\;level\;value\right)}{Total\;number\;of\;surveys\times The\;most\;severe\;stage\;of\;illness}\times 100$$

### 5.7. Statistical Analysis

Statistical analysis was performed using SPSS 26.0 software. One-way analysis of variance (ANOVA) was conducted, followed by Tukey and Duncan tests for multiple comparisons to assess the significance of differences. The significance level was set at *p* < 0.05.

## Data Availability

Data are contained within the article or Supplementary Material.

## Supplementary Materials

The following supporting information can be downloaded at https://www.mdpi.com/article/10.3390/biology13110940/s1. Table S1. Microbial isolates from the healthy and diseased plants.
