# Supplementary material for: Microbial Community of Wilted Fritillaria ussuriensis and Biocontrol Effects of Bacillus tequilensis and Trichoderma koningiopsis
Source: Biology (Basel). 2024 Nov 17;13(11):940. doi: 10.3390/biology13110940 (PMC11592321; doi:10.3390/biology13110940)
Supplement: Supplementary file 1 [file biology-13-00940-s001.zip › biology-3278206-supplementary.pdf]

Table S1. Microbial isolates from the healthy and diseased plants.

Microorganisms (Isolating Source)	Isolate ID	Closest species
Fungi (From diseased plants)	IFM-1, IFM-22, IFM-27, IFM-33, IFM-35, IFM-39, IFM-43, IFM-44, IFM-70, IFM-72, IFM-73, IFM-78, IFM-79, IFM-80, IFM-82, IFM-85, IFM-88	*Fusarium oxysporum*
	IFM-31, IFM-37	*Fusarium redolens*
	IFM-25, IFM-26, IFM-28, IFM-29, IFM-30, IFM-52, IFM-75	*Fusarium solani*
	IFM-45, IFM-49, IFM-50, IFM-51, IFM-69, IFM-71, IFM-74, IFM-81, IFM-84, IFM-87, IFM-89	*Fusarium verticillioides*
	IFM-90	*Fusarium proliferatum*
	IFM-3, IFM-6, IFM-13, IFM-14, IFM-16	*Mucor hiemalis*
	IFM-4, IFM-7, IFM-11, IFM-12, IFM-15, IFM-17, IFM-19, IFM-20, IFM-21, IFM-2, IFM-24, IFM-77	*Mucor circinelloides*
	IFM-18	*Mucor racemosus*
	IFM-10	*Trichoderma harzianum*
	IFM-23	*Trichoderma koningii*
	IFM-46, IFM-83	*Trichoderma hamatum*
	IFM-47	*Trichoderma koningiopsis*
	IFM-48	*Trichoderma longibrachiatum*
	IFM-53, IFM-54, IFM-67, IFM-68，IFM-76	*Trichoderma* sp.
	IFM-55	*Chaetomium elatum*
	IFM-56, IFM-57, IFM-58, IFM-59, IFM-61, IFM-64	*Chaetomium acropullum*
	IFM-5	*Rhizopus oryzae*
	IFM-32, IFM-34, IFM-36, IFM-38, IFM-40, IFM-42, IFM-60	*Bjerkandera adusta*
	IFM-8	*Talaromyces pinophilus*
	IFM-62	*Sistotrema* sp.
	IFM-63, IFM-65	*Irpex lacteus*
	LFM-31, LFM-50, LFM-51	*Bacillus altitudinis*
	LFM-15	*Bacillus anthracis*
	LFM-49	*Bacillus aryabhattai*

Bacteria (From healthy plants)	LFM-27	*Bacillus cereus*
	LFM-24	*Bacillus licheniformis*
	LFM-35	*Lysinibacillus pakistanensis*
	LFM-12, LFM-17, LFM-18, LFM-52	*Bacillus proteolyticus*
	LFM-8, LFM-10, LFM-19, LFM-32, LFM-33, LFM-37, LFM-47	*Bacillus pseudomycoides*
	LFM-11, LFM-13	*Bacillus safensis*
	LFM-1, LFM-23, LFM-25, LFM-38	*Bacillus siamensis*
	LFM-9	*Bacillus sonorensis*
	LFM-39	*Bacillus subtilis*
	LFM-5, LFM-20, LFM-21, LFM-29, LFM-30, LFM-41, LFM-42	*Bacillus tequilensis*
	LFM-2, LFM-7, LFM-14, LFM-26, LFM-28, LFM-55	*Bacillus toyonensis*
	LFM-40	*Bacillus tropicus*
	LFM-4	*Bacillus velezensis*
	LFM-3, LFM-6, LFM-22, LFM-34, LFM-36	*Bacillus wiedmannii*
	LFM-44, LFM-46	*Bacillus zanthoxyli*
	LFM-16, LFM-48	*Bacillus zhangzhouensis*
	LFM-43	*Burkholderia ubonensis*
	LFM-45	*Chryseobacterium sediminis*
	LFM-54	*Chryseobacterium viscerum*
	LFM-53	*Streptomyces lactacystinicus*
Fungi (From healthy plants)	PFM-27	*Clonostachys rosea*
	PFM-23	*Clonostachys* sp.
	PFM-20	*Fusarium acuminatum*
	PFM-2, PFM-3, PFM-6, PFM-8, PFM-12, PFM-17, PFM-22, PFM-37	*Fusarium oxysporum*
	PFM-21	*Fusarium solani*
	PFM-4, PFM-7, PFM-16, PFM-18, PFM-25	*Fusarium verticillioides*
	PFM-5	*Humicola grisea*
	PFM-1, PFM-9, PFM-10, PFM-11, PFM-13, PFM-14, PFM-15, PFM-31, PFM-36	*Penicillium citrinum*
	PFM-33, PFM-34, PFM-35	*Penicillium raperi*
	PFM-24	*Talaromyces pinophilus*
	PFM-19, PFM-26, PFM-29,	*Talaromyces veerkampii*

	PFM-32, PFM-38	
	PFM-28	*Trichoderma longibrachiatum*
	PFM-30	*Trichoderma* sp.
